# Extracts and Abstracts

**Published:** 1885-11

**Authors:** 


### EXTRACTS AND ABSTRACTS.

---

TYROTOXICON,—CHEESE POISON. ABSTRACT OF A PAPER BY PROFESSOR VICTOR C. VAUGHAN, M. D., PH. D.

At a meeting of the Michigan State Board of Health, July 14, 1885, Professor Vaughan presented a report of his investigations on poisonous cheese. It is well known that cases of severe illness follow the eating of some cheese. Such instances are of frequent occurrence in the North German countries and in the United States. In England they are less frequently observed; in France, where much cheese is made and eaten, these cases are said to occur very rarely. A few years ago, the reputation of a large cheese factory in Northern Ohio was destroyed by the great number of cases of alarming illness arising from eating its cheese. Dairymen know this cheese as "sick" cheese.

KINDS OF CHEESE THAT ARE POISONOUS.

A German author says: "The numerous kinds of soft cheese prepared in small families, or on small farms, are generally the cause of the symptoms; while it is quite exceptional to hear of symptoms arising from the use of cheese prepared in large quantities."* Some two years ago a family in Alpena, Michigan, was poisoned by eating cottage cheese; but the cheese which poisoned so many in this State last year, was made at one of the largest factories in the State, and by a thoroughly experienced cheese-maker. The old, foul-smelling cheese, such as Limburger and Schweitzer, have never been known to be poisonous.

EFFECTS OF THE CHEESE.

The symptoms produced by "sick" cheese, as reported by German and American physicians, agree quite closely and are as follows: Dryness of the mouth and throat with a sense of constriction, nausea, vomiting, diarrhœa, headache, sometimes double vision, and marked nervous prostration. In rare instances the sufferer dies from collapse. As a rule, recovery occurs in a few hours, or at most after a few days. The symptoms of cheese-poisoning and those of sausage, canned-meat and fish-poisoning are very similar, though death results more frequently from the others mentioned than from cheese-poisoning.

APPEARANCE OF THE CHEESE.

The samples of poisonous cheese examined had no peculiarities of appearance, odor, or taste, by which they could be distinguished from good cheese. It is true that if two pieces of cheese — one poisonous and the other wholesome — were offered to a dog or a cat, the animal would select the good cheese. But this would be probably due to an acuteness of the sense of smell possessed by the animal and not belonging to man. Indeed, if a person tasted a cheese knowing that it was poisonous, he might detect a sharpness of taste which would not ordinarily be noticed.

HAVE WE ANY READY MEANS OF RECOGNIZING POISONOUS CHEESE.

There is no certain means, aside from a chemical examination, by which a poisonous cheese can be distinguished from a wholesome one. The most reliable ready method is probably that proposed by Professor Vaughan a year ago, and it is as follows: Press a small strip of blue litmus paper against a freshly cut surface of the cheese; if the paper is reddened instantly and intensely, the cheese may be regarded with suspicion. When treated in this way any green cheese will redden the litmus paper, but ordinarily the reddening will be produced slowly and will be slight. If the piece of cheese be dry, a small bit should be rubbed up with an equal volume of water, and the paper should then be dipped in the water. Professor Vaughan does not regard the above test as free from error, but as the most reliable and ready means now known. Every grocer should apply this test to each fresh cheese which he cuts. The depth of the reddening of the paper may be compared with that produced by cheese which is known to be wholesome.

### EFFECTS ON THE LOWER ANIMALS.

Dogs and cats, at least, are not affected by eating poisonous cheese. This is probably due to the fact that they do not get enough of the poison from the amount of cheese which they eat. The pure isolated poisons in sufficient doses would undoubtedly produce upon the lower animals effects similar to those produced on man.

### NATURE OF THE POISON.

Professor Vaughan has succeeded in isolating the poison, to which he has given the name tyrotoxicon (from two Greek words, which mean cheese acid poison). It is the product of slight putrefaction in the cheese, which probably occurs in the vat, as the curd has been known to poison a person. By this slight putrefaction, or excessive fermentation, as it may be called, a large amount of butyric acid is formed, and this in the presence of the casein of the cheese is capable of developing the poison. Different samples of the poisonous cheese contain different amounts of the poison. The same weight of cheese from one cake furnished three times as much poison as that from another cake. The poison was obtained in long, needle-shaped crystals, which are freely soluble in water, alcohol, chloroform and ether. The smallest visible fragment of a crystal placed on the tongue causes a sharp, stinging pain at the point of application, and, in a few minutes, dryness and constriction of the throat. A slightly larger amount produces nausea, vomiting and diarrhœa. The poison is volatile at the temperature of boiling water, and for this reason even poisonous cheese may be eaten with impunity after being cooked. The substance has also a marked, pungent odor, and through the nose one can obtain sufficient of the volatile poison to produce dryness of the throat. This is true, however, only of the isolated poison. In the cheese the taste and odor of the poison are both modified to such an extent that they would not be recognized, as has already been stated.

The first step in the study of cheese poisoning has now been taken, by finding out what the poison is. Efforts will be made to ascertain the means for preventing its formation.

---

EXTRA-PERITONEAL RESECTION OF THE BLADDER FOR CARCINOMA. *By* G. v. AUTAL, *reported in Centralblatt für Chirurgie. Translated by* MARY E. BATES, M. D., *of Chicago.*

The patient was a male, aet. 61. There was dysuria for two years. The urine was bloody with blood-clots for one year. There was micturition every ten to twelve minutes,—a few drops of bloody urine, followed by blood-coagula passed with great pain. Hæmorrhage from bladder was continuous, day and night, and not affected by rest or motion. The patient was emaciated and weakened. His skin was a dirty dun-color.

*Combined Examination.*—A tumor was found, of fist-size, apparently inserted in the summit of the bladder, with an uneven surface in the bladder-wall, which was stony hard in places, and gave the sound of stone when struck.

The bladder was freely movable. There was no infiltration of glands.

The specific gravity of the urine was 1027. Its reaction was alkaline. Two-thirds of the sediment was coagulated blood.

*Microscopical examination.*—There were blood corpuscles in great numbers. There was much pus and also bladder-epithelium. No organized tissue or kidney-elements found.

*Diagnosis.—Incrusted tumor of the summit of the bladder.* —The differential diagnosis between that and stone (encapsuled) was based upon the continuance of the hæmorrhage even after many days of rest of the patient.

Operation, April 23, 1885.

After thorough disinfecting irrigation, the bladder was filled with 250 ccm. of salt water and a colpeurynter placed in the rectum.

On opening the abdominal wall the antevesical fold of peritoneum was found to extend down so far that between it and the pubes there was a space of but 1 cm. in breadth. In this space that part of the bladder uncovered by peritoneum appeared. The antevesical peritoneal fold was carefully pushed up and held, and the bladder opened by a beveled incision 4 cm. long.

Two fingers were passed through this opening, and an incrusted, friable, uneven tumor, in intimate connection with all of the coats of the bladder, was found in its summit.

Owing to its cancer-like nature, it was decided to remove the entire thickness of the bladder-wall to an extent corresponding to the base of the tumor, that is, to resect that much of the bladder.

First—The peritoneum was carefully separated from the summit, and in part from the posterior wall of the bladder, pushed up and held by an assistant.

Second—The summit was encircled and removed by an incision, so made that the edges of the bladder-wound presented the appearance of a shallow funnel. One-third of the entire bladder was removed. Seven bleeding vessels were ligated with sublimated silk ligature cut short.

Third—Toilet of the bladder and the wound was made.

Fourth—The wound of bladder was closed with sublimated silk sutures.

Fifth—Two hard rubber " knee "-shaped tubes were placed in the lower angle of the wound, through which the bladder was to be continually irrigated with a solution of thymol.

S xth—The abdominal wound was closed by sublimated silk sutures, except at the lower angle.

On the 6th day, irrigation was stopped; a Nélaton catheter was introduced into the bladder *per urethram*, and a long drainage tube through the abdominal wound. The greater part of the urine escaped through this wound. On the 12th day the drainage tube was removed. The granulations around the fistula-opening were curetted, and by June 17 the opening had closed completely. The convalescence was fever-free. The patient was much improved he could retain his urine for three or four hours. It was no longer bloody.

The bladder-capacity, 200 to 300 ccm.

NOTE.—According to v. Autal there have been but two partial resections of the bladder made. The first by Sonnenburg who resected two-thirds of the bladder, including the peritoneal covering, and closing the remaining part of the bladder by uniting it with the edges of the peritoneal wound. The patient died in four weeks, from exhaustion. The second—the case reported above—presented two modifications of Sonnenburg's operation and of similar expermental partial resections of the bladders of animals, (by Maximow, Vincent, Snamensky). The first modification, and the more important one, consisted in the separating and holding up of the peritoneal covering of the bladder, thereby preventing the opening into the peritoneal cavity, and the consequent dangers of infection from the decaying ulcerating tumor. The second modification is the beveled-incision wound. This in the coaption of the wound, favors the prevention of infiltration of urine, especially necessary at the upper and back part, where the peritoneum has been separated from the bladder.

---

PARALYSIS OF THE BLADDER FROM THE USE OF CARBOLIC ACID.

Cartaz, in the *Gazette méd. de Paris*, 1884, reports interesting cases of vesical paralysis due to the use of carbolic acid in surgical dressings. This is one of the various toxic effects of the acid, and has been observed by the writer only twice, occurring in the first instance after irrigation of the uterus with a two per cent. solution of carbolic acid after abortion. The paralysis disappeared on the substitution of corrosive sublimate as a disinfectant. The second instance was met with in the case of an aged woman with a fracture of the neck of the femur. The dressings, which were daily changed, were treated with a five per cent. solution of carbolic acid. In two weeks there was enormous distension of the bladder, and for forty-eight hours the woman had not urinated. By the catheter about a quart of dark-brown urine was evacuted. In four days the paralysis disappeared, the carbolic solution having been replaced by boracic acid ointment. There appeared no reason to doubt that in each case the paralysis of the bladder was caused by the drug in question, as in both patients the urine had the colour characteristic of carbolic acid poisoning, and the symptoms of paralysis in each case disappeared on abandoning the use of the drug. Only two similar cases are reported, and in these, vesical paralysis resulted from drinking a concentrated solution of the acid.—*Centralb. für Chirurgie*, March 28, 1885.

TURPENTINE IN DIPHTHERIA.

Dr. N. Lunin, of St. Petersburg, in a recent contribution to the *Med. Wochensch.*, presents the results of a study of two hundred and ninety-six cases of diphtheria, classified, with reference to treatment, into the fibrinous and the phlegmonous-septic forms. The treatment of the first form with corrosive sublimate, iron and chinolin resulted in a death-rate of 30, 32 and 31 per cent., respectively. The administration of oil of turpentine in ten-drop doses every hour was followed by the recovery of 91 per cent. of twenty-three cases. In the phlegmonous-septic form the mortality was slightly less under the iron treatment. His tables indicate an average death-rate of 55.3 per cent., and will no doubt disgust a large number of medical practitioners and bacteriologists who are happy in the belief that they have found a specific for diphtheria.

---

DR. EDWARD N. LIELL reports, *New York Medical Journal*, September 19th, an interesting case of caffeine poisoning. The condition of the patient is thus described: "Greatly prostrated and exhausted, semi-unconscious, cold extremities, clammy perspiration, anæsthesia and slight paresis of the muscles of both feet; temperature normal, pulse 55 and irregular, respiration irregular and 16 per minute. Under treatment by revulsives and administration, hypodermically, of $\frac{1}{50}$ gr. atropin sulph. and diffusible stimulants, prompt improvement was effected, but the toxic effect continued for five days, characterized by abdominal pain, dimness of vision, delirium, vertigo, muscular tremors, difficulty in deglutition and speech, increased action of kidneys, frequent urination, and tetanic convulsions. The quantity taken amounted to eighteen grains within a period of an hour and a half. The treatment was successful, consisting chiefly in the employment of chloral-hydrate and potassium bromide in liberal doses.

